# Repeat endoscopic endonasal transsphenoidal surgery for residual or recurrent Cushing’s disease: safety, feasibility, and success

**DOI:** 10.1007/s11102-024-01396-x

**Published:** 2024-05-15

**Authors:** Sahin Hanalioglu, Muhammet Enes Gurses, Neslihan Nisa Gecici, Baylar Baylarov, Ilkay Isikay, Alper Gürlek, Mustafa Berker

**Affiliations:** 1 Department of Neurosurgery, Faculty of Medicine, Hacettepe University, Ankara, Turkey; 2 Department of Neurosurgery, Miller School of Medicine, University of Miami, Miami, FL USA; 3 Department of Endocrinology and Metabolism, Faculty of Medicine, Hacettepe University, Ankara, Turkey; 4 Department of Neurosurgery, Hacettepe University School of Medicine, Sihhiye, Ankara 06230 Turkey

**Keywords:** Cushing’s disease, Pituitary adenoma, ACTH, Corticotropinoma, Endoscopic surgery, Recurrence, Reoperation

## Abstract

**Purpose:**

The success and outcomes of repeat endoscopic transsphenoidal surgery (ETS) for residual or recurrent Cushing’s disease (CD) are underreported in the literature. This study aims to address this gap by assessing the safety, feasibility, and efficacy of repeat ETS in these patients.

**Methods:**

A retrospective analysis was conducted on 56 patients who underwent a total of 65 repeat ETS performed by a single neurosurgeon between January 2006 and December 2020. Data including demographic, clinical, laboratory, radiological, and operative details were collected from electronic medical records. Logistic regression was utilized to identify potential predictors associated with sustained remission.

**Results:**

Among the cases, 40 (61.5%) had previously undergone microscopic surgery, while 25 (38.5%) had prior endoscopic procedures. Remission was achieved in 47 (83.9%) patients after the first repeat ETS, with an additional 9 (16.1%) achieving remission after the second repeat procedure. During an average follow-up period of 97.25 months, the recurrence rate post repeat surgery was 6.38%. Sustained remission was achieved in 48 patients (85.7%), with 44 after the first repeat ETS and 4 following the second repeat ETS. Complications included transient diabetes insipidus (DI) in 5 (7.6%) patients, permanent (DI) in 2 (3%) patients, and one case (1.5%) of panhypopituitarism. Three patients (4.6%) experienced rhinorrhea necessitating reoperation. A serum cortisol level > 5 µg/dL on postoperative day 1 was associated with a reduced likelihood of sustained remission.

**Conclusion:**

Repeat ETS is a safe and effective treatment option for residual or recurrent CD with satisfactory remission rates and low rates of complications.

**Supplementary Information:**

The online version contains supplementary material available at 10.1007/s11102-024-01396-x.

## Introduction

Cushing’s disease (CD) arises from an adrenocorticotropic hormone (ACTH)-secreting pituitary adenoma, leading to excessive endogenous glucocorticoid production [1]. The reported incidence of CD varies from 0.7 to 2.4 cases per million individuals annually [2–6]. Hypercortisolism impacts every bodily system and is linked to elevated morbidity and mortality risks [7, 8]. Therefore, prompt CD diagnosis and management are crucial to enhance patient outcomes.

Transsphenoidal surgery remains the primary treatment for CD, and have been associated with satisfactory remission rates ranging from 65 to 94% [2, 3, 5, 9–11]. Two surgical techniques are utilized: microscopic and endoscopic approaches. While both methods are effective, studies indicate that endoscopic transsphenoidal surgery (ETS) offers higher rates of complete tumor removal and lower complication rates [12–14]. ETS holds advantages over microscopic transsphenoidal surgery (MTS) due to superior tumor visualization, especially for laterally invasive tumors and macroadenomas [15]. Since its introduction in 1997, ETS has gained popularity and is now the standard surgical approach for managing CD [16].

Remission rates post-ETS for CD treatment range from 77 to 90% [17–22]. Despite ETS’s technical benefits and favorable outcomes, recurrence rates for Cushing’s disease after successful ETS range between 5.6% and 22.8% [17, 18, 22, 23]. Reoperating for residual or recurrent CD presents challenges due to altered surgical landmarks and scar tissue formation from previous surgeries, potentially elevating morbidity, and mortality risks [24, 25]. Limited literature exists on the success and outcomes of repeat endoscopic transsphenoidal surgery for residual or recurrent CD. This study aims to address this gap by assessing the safety, feasibility, and efficacy of repeat ETS in patients with residual or recurrent Cushing’s disease.

## Methods

### Study design

This is a retrospective cohort study of repeat endoscopic transsphenoidal surgery for residual or recurrent Cushing’s disease. All patients underwent endoscopic endonasal transsphenoidal surgery by the senior author between 2006 and 2020. The study protocol was approved by the local ethics committee for clinical studies.

### Patient selection

The study participants were selected based on specific inclusion and exclusion criteria. Inclusion criteria were as follows: (i) a confirmed diagnosis of Cushing’s disease, (ii) prior transsphenoidal surgery, and (iii) confirmation of residual or recurrent CD through clinical, laboratory, and/or imaging assessments. Exclusion criteria included: (i) prior craniotomy without transsphenoidal surgery, (ii) previous radiotherapy before reoperation, (iii) inaccessible clinical, laboratory, or radiological data, and (iv) follow-up duration of less than 6 months.

### Diagnostic criteria

Each patient underwent thorough screening for active Cushing’s disease. An increased 24-hour urine cortisol level > 45 µg/day or a serum fasting cortisol level exceeding 1.8 µg/dl following a low-dose (2 mg) dexamethasone suppression test was deemed abnormal. Subsequently, a high-dose (8 mg) dexamethasone test was administered, and a reduction of 50% or more from the baseline value was indicative of active Cushing’s disease. Due to the technical limitations of the institution that the research has been done, late-night salivary cortisol tests were not performed. Early remission was characterized by a fasting serum cortisol level below 5 µg/dl on the 1st and 7th postoperative days. Patients displaying a serum cortisol level below 1.8 µg/dl after the low-dose dexamethasone suppression test or those requiring continued corticosteroid replacement post-surgery were considered to maintain remission. The presence of a residual adenoma on postoperative magnetic resonance imaging (MRI) confirmed residual disease.

### Routine follow-up protocol

Patients were evaluated for Cushing’s disease symptoms before surgery and monitored at 6 months after surgery, as well as during yearly check-ups for any changes in their condition. Fasting serum ACTH and cortisol levels were measured in the morning before surgery, on the 1st and 7th days after surgery, at the 1st, 3rd, and 6th months, and during yearly follow-up appointments. Prior to surgery, all patients underwent contrast-enhanced pituitary MRI and paranasal sinus CT scans. Follow-up pituitary MRI scans were conducted on the 1st day, at 3 and 12 months after surgery, and then annually thereafter.

### Data collection

Data from electronic medical records were gathered, encompassing demographic, clinical, laboratory, radiological, and operative details. Laboratory assessments comprised an anterior pituitary hormone panel (Follicle-stimulating hormone [FSH], Luteinizing hormone [LH], Thyroid-stimulating hormone [TSH], Prolactin [PRL], Growth hormone [GH]), serum electrolytes, preoperative and postoperative serum ACTH, and cortisol levels. Patient records, along with CT and MRI scans, were scrutinized to document preoperative tumor characteristics such as size, multifocality, relationship with the cavernous sinus, Hardy-Wilson classification of sellar destruction, and suprasellar extension. Tumors larger than 10 mm were classified as macroadenomas. The operative database was examined to collect data on previous surgeries, including the number and dates of prior procedures, as well as the surgical techniques utilized. Outcome measures comprised remission rates and surgical complications.

### Statistical analysis

Statistical analysis was conducted utilizing SPSS 23.0 software (IBM, New York). Two-group comparisons were performed using Chi-square and Fisher’s exact tests for categorical variables and Student’s t-test for continuous variables. Categorical variables were presented as numbers and percentages, while continuous variables were presented as means ± SD or median [IQR]. Logistic regression was performed to investigate potential predictors linked to sustained remission. A p-value of < 0.05 was deemed statistically significant.

## Results

### Baseline characteristics

Supplementary File 1 displays the demographic characteristics of the patient cohort.

A retrospective analysis was conducted on 190 patients who underwent a total of 212 operations for CD at our department between January 2006 and December 2020. Among them, 56 patients, comprising 65 repeat endonasal transsphenoidal surgeries due to either recurrence (*n* = 18, 27.7%) or residual disease (*n* = 47, 72.3%), were identified. The majority of patients were female (*n* = 48, 85.7%), with a mean age of 37.6 ± 12.4 years. Of the 56 patients, 43 (76.8%) were referred from another institution. Most patients (*n* = 42, 75%) had undergone only one prior surgery, while 12 patients (21.4%) had a history of two previous surgeries, and 2 patients (3.6%) had undergone three prior surgeries before referral to our center. The average follow-up duration since the first repeat ETS was 97.2 ± 36.8 months. The mean time to recurrence was 80.2 ± 61.1 months (median 75 months, range 23.2 to 103.5 months).

### Hormonal data

Table 1 depicts the preoperative and postoperative serum ACTH and cortisol levels. The average preoperative serum cortisol levels for the entire patient cohort stood at 18.7 ± 11.1 µg/dL (median 17, range 12-24.6). The median preoperative 24-hour urine free cortisol level was 237 µg /day [188.5–425.5]. On the initial postoperative day, the mean serum cortisol levels for all patients were 13.4 ± 13.8 µg/dL (median 6.4, range 1.7–21). In 46.2% of cases (*n* = 30), cortisol levels on the first postoperative day were below 5 µg/dL (< 2 µg/dL in 33.8%). A comparison of the mean preoperative and postoperative serum ACTH and cortisol levels between the groups with residual disease and recurrence is detailed in Table 1.

**Table 1:** Cohort overview and comparison of recurrence and residual disease groups

Variable	Total (*n* = 65)	Residual Disease (*n* = 47)	Recurrence (*n* = 18)	*p*-value
Technique of the previous surgery				**< 0.001**
MTS	40 (61.5)	36 (76.6)	4 (22.2)	
ETS	25 (38.5)	11 (23.4)	14 (77.8)	
Tumor size				
Microadenoma	41 (63.1)	30 (63.8)	11 (61.1)	0.839
Macroadenoma	24 (36.9)	17 (36.2)	7 (38.9)	
Multifocality				
Unifocal	50 (76.9)	37 (78.7)	13 (72.2)	0.743
Bifocal	15 (23.1)	10 (21.3)	5 (27.8)	
Relation to cavernous sinus				
Extension	21 (32.3)	15 (31.9)	6 (33.3)	0.589
Invasion	10 (15.4)	6 (12.8)	4 (22.2)	
No relationship	34 (52.3)	26 (55.3)	8 (44.4)	
Hardy-Wilson Classification				0.339
Grade				
I	38 (58.5)	25 (59.5)	8 (57.1)	
II	16 (24.6)	8 (19)	5 (5)	
III	6 (9.2)	6 (14.3)	1 (7.1)	
IV	5 (7.7)	3 (7.1)	0 (0)	
Stage				0.443
A	30 (46.2)	19 (45.2)	7 (50)	
B	7 (10.8)	4 (9.5)	3 (21.4)	
C	2 (3.1)	2 (4.8)	0 (0)	
D	1 (1.5)	0 (0)	0 (0)	
E	25 (38.5)	17 (40.5)	4 (28.6)	
Laboratory values				
Preoperative serum ACTH (pg/mL)	182.71 ± 577.08 60.5 [37.15–104.5]	220.7 ± 675.73	83.5 ± 61.7	0.395
Preoperative serum Cortisol (µg/dL)	18.75 ± 11.16 17 [12-24.65]	19.18 ± 12.11	17.64 ± 8.39	0.621
Postoperative serum ACTH (pg/mL)	43.29 ± 50.2 25.5 [15.8–53.7]	43.07 ± 45.42	43.94 ± 63.96	0.953
Postoperative serum Cortisol (µg/dL)	13.41 ± 13.85 6.45 [1.77–21.01]	14.62 ± 14.52	10.25 ± 11.7	0.259
POD 1 Cortisol level				0.700
>5 µg/dL	35 (53.8)	26 (55.3)	9 (50)	
≤5 µg/dL	30 (46.2)	21 (44.7)	9 (50)	
Tumor pathology				0.198
ACTH + adenoma	55 (85)	40 (85.1)	15 (83.3)	
Crooke degeneration	2 (3)	1 (2.1)	1 (5.6)	
Pituitary hyperplasia	2 (3)	1 (2.1)	1 (5.6)	
Normal pituitary tissue	6 (9)	5 (10.6)	1 (5.6)	
Result of reoperation				0.740
Remission	51 (78.5)	36 (76.6)	15 (83.3)	
Residual disease	14 (21.5)	11 (23.4)	3 (16.7)	

Values are shown as number (%), mean ± SD or median [IQR] unless otherwise indicated

*Abbreviations* MTS, microscopic transsphenoidal surgery; ETS, endoscopic transsphenoidal surgery; ACTH, adrenocorticotropic hormone; POD 1, postoperative day 1

### Radiological findings

In the entire case cohort, there were 41 microadenomas (63.1%) and 24 macroadenomas (36.9%). Fifteen cases (23.1%) exhibited bifocal adenomas. Adenoma extension into the cavernous sinuses, indicated by cavernous sinus wall displacement, was present in 21 cases (32.3%), while invasion into the cavernous sinuses was observed in 10 cases (15.4%). Based on the Hardy-Wilson Classification, there were 38 Grade I adenomas (58.5%), 16 Grade II adenomas (24.6%), 6 Grade III adenomas (9.2%), and 5 Grade IV adenomas (7.7%). Thirty patients (46.2%) presented with Stage A adenoma, 7 (10.8%) with Stage B adenoma, 2 (3.1%) with Stage C adenoma, 1 (1.5%) with Stage D adenoma, and 25 (38.5%) with Stage E adenoma. As indicated in Table 1, there were no statistically significant differences between patients with residual disease and recurrence concerning radiological findings.

### Surgical characteristics

A single surgeon conducted all 65 reoperations. Among these, 47 patients (72.3%) underwent repeat ETS due to residual disease, while 18 (27.7%) did so due to recurrence. The previous surgical technique was microscopic in 40 cases (61.5%) and endoscopic in 25 cases (38.5%). Microscopic transsphenoidal surgeries were exclusively performed at other institutions. There was a notable disparity between patients with residual disease and recurrence regarding the technique of the previous surgery. Residual disease occurrence following endoscopic transsphenoidal surgery was less frequent (*n* = 11/25, 44%) compared to after microscopic transsphenoidal surgery (*n* = 36/40, 90%; *p* < 0.001) (Table 1). Immunohistochemical staining of the specimens indicated that 55 cases (85%) exhibited ACTH-positive adenoma. Nevertheless, all patients with a negative pathology at the repeat surgery had a confirmed ACTH-adenoma at the first surgery. Of the 10 patients (15%) with a negative ACTH-positive adenoma pathology, two patients underwent inferior petrosal sinus sampling (IPSS) previously and were confirmed to have CD. Remaining patients did not undergo an additional inferior petrosal sinus sampling (IPSS) because all functional test results indicated a central source and MRI confirmed pituitary microadenoma in all cases. Notably, there are studies reporting that IPSS may not be required in patients with a sellar mass and a biochemical testing suggestive of CD [26, 27]. Additionally, we also explored both sides of the pituitary and confirmed the adenoma intraoperatively. Therefore, negative pathology in the repeat surgery is most likely due to sampling error.

### Outcomes

**Fig. 1:**
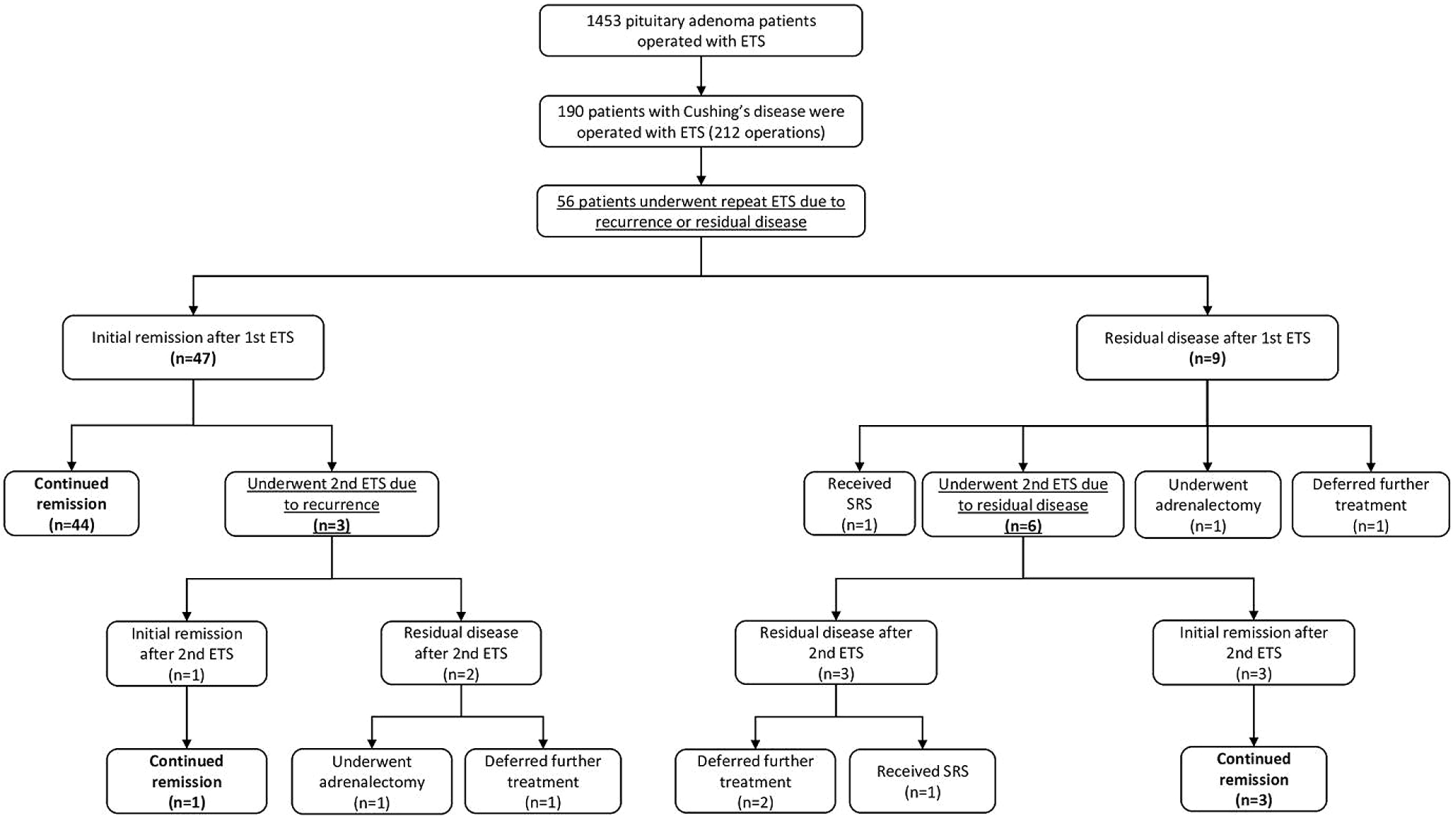
Outcomes of repeat endoscopic transsphenoidal surgery for residual or recurrent Cushing’s disease

As depicted in Fig. 1, among the 56 patients, 47 (83.9%) experienced initial remission following the first repeat ETS, while 9 (16.1%) still had residual adenoma. Within the group achieving initial remission, 44 patients (93.6%) maintained remission without the need for further surgeries, while 3 (6.4%) experienced recurrence during follow-up and required a second repeat ETS.

**Fig. 2:**
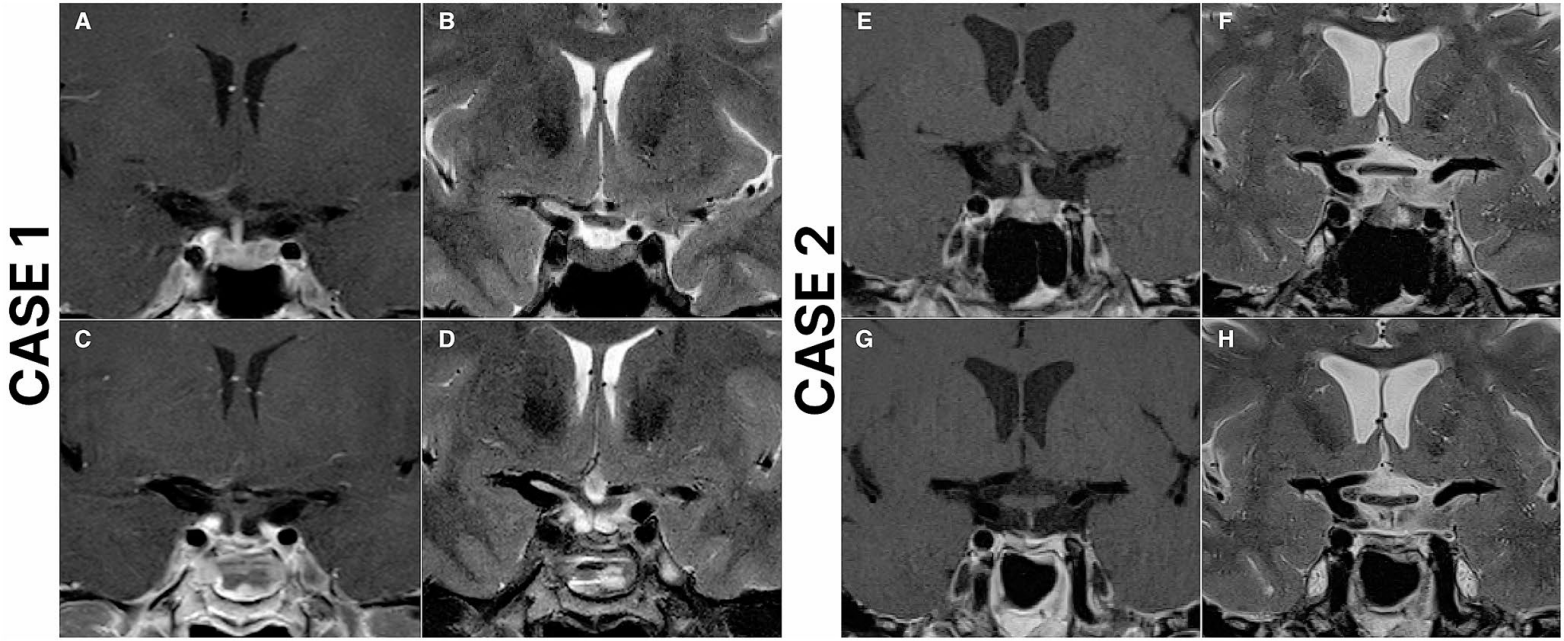
Case 1: Preoperative and postoperative magnetic resonance imaging (MRI) scans of a 49-year-old female who underwent repeat endoscopic transsphenoidal surgery (ETS) due to recurrent Cushing’s disease and achieved remission. The patient underwent initial surgery 14 years ago at an outside institution. Preoperative T2 (**A**), and T1 contrast-enhanced (**B**) MRI scans demonstrate a right-sided pituitary adenoma. Postoperative T2 (**C**), and T1 contrast-enhanced (**D**) MRI scans demonstrate total resection of the adenoma. Case 2: Preoperative and postoperative magnetic resonance imaging (MRI) scans of a 53-year-old female who underwent repeat endoscopic transsphenoidal surgery (ETS) due to recurrent Cushing’s disease and achieved remission. The patient underwent initial surgery 3 years ago at an outside institution. Preoperative T2 (**E**), and T1 contrast-enhanced (**F**) MRI scans demonstrate a left-sided pituitary adenoma, in close relation to ICA. Postoperative T2 (**G**), and T1 contrast-enhanced (**H**) MRI scans demonstrate total resection of the adenoma

Among the 9 patients with residual disease after the first repeat ETS, 1 (11.1%) opted to defer further treatment, 1 (11.1%) received radiotherapy, 1 (11.1%) chose adrenalectomy, and 6 (66.7%) underwent a second repeat ETS. Of the 9 patients who underwent a second repeat ETS due to residual disease or recurrence, 4 (44.4%) sustained remission, 5 (55.6%) still had residual disease, but 3 of them deferred further treatment, 1 received radiotherapy, while 1 achieved remission after adrenalectomy. Overall, 78.5% (*n* = 51) of the entire case cohort achieved remission following repeat ETS. Representative cases are presented in Fig. 2.

Transient diabetes insipidus (DI) developed in 5 patients (7.6%), while 2 (3%) experienced permanent DI following repeat ETS. Intraoperative cerebrospinal fluid (CSF) leak occurred in 20 operations (30.7%). Three patients (4.6%) developed rhinorrhea and required reoperation. Five patients (7.6%) developed prolactin deficiency, 3 patients (4.6%) had GH deficiency, and another 3 patients (4.6%) had TSH deficiency requiring thyroxine replacement. Four patients (6.2%) had combined deficiencies in TSH, FSH, LH and prolactin, while one patient (1.5%) developed panhypopituitarism following the second repeat ETS.

### Factors predisposing to unsuccessful repeat endoscopic transsphenoidal surgery

Among the 42 patients who underwent repeat ETS for residual disease, 9 (21.4%) still had residual disease after the first repeat ETS. We conducted a multivariable logistic regression analysis to explore potential risk factors for unsuccessful repeat ETS. However, the analysis did not reveal any significant association between the success of repeat ETS and factors such as extension or invasion into cavernous sinuses, sellar or parasellar extension, or tumor size (Supplementary File 1).

### Potential predictors of sustained remission

We conducted a multivariable logistic regression analysis to investigate possible predictors of sustained remission. The variables included in the analysis are detailed in Table 5. The results indicated that having a serum cortisol level exceeding 5 µg/dL on postoperative day 1 was linked to a decreased likelihood of achieving sustained remission (Odds ratio [OR] 0.09, 95% confidence interval [CI] 0.01–0.52, *p* = 0.006) (Table 2).

**Table 2:** Logistic regression analysis of potential predictors for continued remission

Variable	OR (95% CI)	*p*-value
Age	1.003 (0.94–1.06)	0.913
Gender		
Female	Reference	
Male	0.43 (0.06–2.88)	0.387
Indication for repeat ETS		
Residual disease	Reference	
Recurrence	1.2 (0.25–5.68)	0.812
Tumor size		
Microadenoma	Reference	
Macroadenoma	0.94 (0.18–4.79)	0.948
Relation to cavernous sinus		
No relation	Reference	
Extension-Invasion	0 (0)	0.999
Hardy-Wilson Classification		
Grade		
I-II	Reference	
III-IV	3.2 (0.3-34.06)	0.334
Stage		
A-C	Reference	
D-E	0 (0)	0.999
POD 1 Cortisol level		
≤5 µg/dL	Reference	
>5 µg/dL	0.09 (0.01–0.52)	**0.006**

*Abbreviations* ETS, endoscopic transsphenoidal surgery; POD 1, postoperative day 1

## Discussion

Transsphenoidal surgery remains the established standard for treating Cushing’s disease, with demonstrated remission rates ranging from 65 to 94%, contingent upon the surgeon’s expertise and remission criteria [2, 3, 5, 9–11]. The advent of endoscopic techniques has notably augmented this approach, offering wider visibility, reduced nasal trauma, and shorter hospital stays [16, 25, 28, 29]. While the effectiveness of ETS in managing CD is well-documented, literature on its efficacy in treating residual or recurrent cases is limited. Our study addresses this gap by assessing the safety, feasibility, and outcomes of repeat ETS for patients with persistent or recurrent Cushing’s disease.

In our study, 56 patients underwent 65 repeat ETS procedures for residual or recurrent Cushing’s disease. Mean follow-up duration was 97.2 ± 36.8 months, which is one of the longest follow-up durations that has been reported following repeat endoscopic transsphenoidal surgery [5, 30–32]. Of these patients, 40 (61.5%) had previously undergone microscopic surgery, while 25 (38.5%) had undergone prior endoscopic procedures. Importantly, a notable difference emerged between patients with residual disease and those experiencing recurrence regarding the prior surgical approach, with residual disease being less frequent after endoscopic surgery compared to microscopic surgery (*p* < 0.001). This variance was expected, as numerous studies have indicated that ETS yields a higher rate of complete resection compared to MTS [12–14].

After the first repeat ETS, 47 patients (83.9%) achieved remission, and 78.5% (*n* = 44) of them maintained remission at a mean follow-up of 97.2 months without requiring additional surgery. Limited data exists regarding the remission rates of CD following repeat transsphenoidal surgery, with reported rates ranging from 28.9 to 73% [33, 34, 35]. Burke et al. reported an immediate remission rate of 86.7% and a continued remission rate of 73.3% at follow-up after repeat ETS [36]. Among our patients who achieved remission after successful repeat ETS, 3 individuals (6.38%, *n* = 3/47) experienced recurrence after the first repeat ETS, with a mean time to recurrence of 45.6 months. The rates of CD recurrence following reoperation vary, with documented rates ranging between 22% and 63.2% [37, 38]. In our study, 9 patients required a second repeat ETS due to residual disease or recurrence. Of these, 4 (44.4%) achieved continued remission following the second repeat ETS, while 5 (55.6%) had residual disease; however, 4 of them deferred further treatment, and 1 achieved remission after adrenalectomy. In total, 47 patients (83.9%) in the entire patient cohort achieved remission following endoscopic transsphenoidal surgery and did not require further intervention.

Within our case cohort, among the 42 patients who underwent repeat ETS for residual disease, 9 individuals (21.4%) continued to exhibit residual disease following the first repeat ETS. We did not establish a significant association between the success of repeat ETS and factors such as extension or invasion into cavernous sinuses, sellar or parasellar extension, or tumor size.

The degree of hypocortisolism following transsphenoidal surgery is considered a potential indicator of remission in the postoperative period [3]. Numerous studies have indicated that patients with subnormal postoperative cortisol levels tend to experience a lower recurrence rate compared to those with normal or supranormal levels, although consensus on the precise cutoff level remains elusive [30–32, 39]. In a retrospective study involving 52 patients with CD, researchers reported a 100% positive predictive value of a postoperative nadir cortisol level < 2 µg/dL for achieving remission [5]. Additionally, Esposito et al. observed that a morning serum cortisol level ≤ 5 µg/dL on postoperative day 1 or 2 appears to serve as a reliable predictor of remission [11]. In our investigation, logistic regression analysis revealed that patients with a serum cortisol level > 5 µg/dL on postoperative day 1 were less inclined to achieve continued remission compared to those with a serum cortisol level < 5 µg/dL on postoperative day 1.

Repeat transsphenoidal surgery presents unique challenges due to distorted surgical landmarks and the presence of scar tissue from prior procedures, often resulting in lower cure rates and increased morbidity risk [24, 25, 28]. Non-surgical options such as radiotherapy and radiosurgery have been considered as an effective treatment option for recurrent or residual CD due to low rates of morbidity and acceptable remission rates [28, 40]. However, our findings suggest that the outcomes and complication rates associated with repeat ETS are comparable to primary ETS for CD and superior to other non-surgical options for residual or recurrent CD. Within our patient cohort, 5 (7.6%) individuals experienced transient diabetes insipidus (DI), while 2 (3%) developed permanent DI. Additionally, one patient (1.5%) experienced panhypopituitarism following the second repeat ETS. Similarly, various studies have reported DI rates ranging from 2 to 13% and panhypopituitarism rates between 2% and 9.7% [25, 28, 41–43]. In our series, 3 (5.3%) patients developed rhinorrhea and required reoperation, consistent with reported rates of postoperative CSF leak ranging from 1 to 5% following repeat endoscopic transsphenoidal surgery for residual or recurrent pituitary tumors [25, 28, 44]. While radiotherapy and radiosurgery are options for patients who have failed transsphenoidal surgery or experienced recurrence, the literature suggests remission rates ranging from 46 to 84%, with several studies indicating high recurrence rates (25-50%) following radiotherapy [40, 45–47]. In our study, among 56 patients, 47 (83.9%) achieved remission following the first repeat ETS, while 4 (17.8%) achieved remission after the second repeat ETS. Over a mean follow-up duration of 97.25 months, our recurrence rate following repeat ETS was 27.7%, with a mean time to recurrence of 45.62 months.

**Fig. 3:**
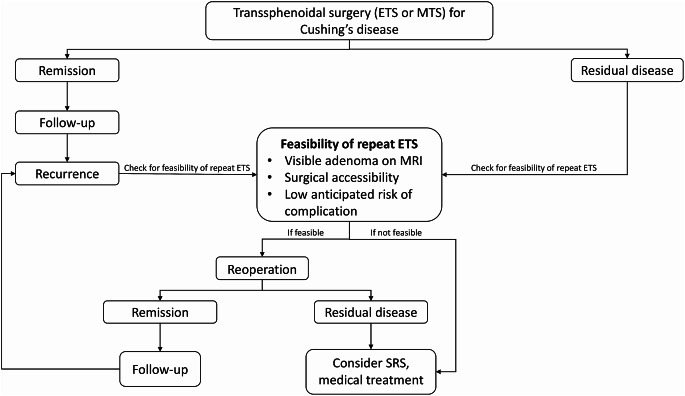
Specific algorithm for the management of Cushing’s disease patients

At our institution, we adhere to a specific algorithm (Fig. 3) for managing Cushing’s disease patients and implement a meticulous protocol for individuals undergoing repeat ETS for residual or recurrent CD. A thorough clinical and radiological assessment is conducted for all patients before surgery. Detailed radiological evaluation is particularly essential to identify any distortions in surgical landmarks from prior procedures, such as the course of sphenoidal septa and the location of the sellar floor opening, as well as other potential aberrations like internal carotid artery and optic nerve dehiscence. Imaging techniques should encompass dynamic pituitary MRI with and without contrast and paranasal CT scans. Our objective is to achieve extensive exposure during surgery, which is especially critical for managing bifocal adenomas or adenomas with cavernous sinus invasion or extension. The expanded visual field also facilitates the visualization of concealed parts of the adenoma, allowing the surgeon to achieve complete resection, which may be challenging or even impossible with limited exposure. We employ a multilayer closure technique to prevent CSF leaks, and if necessary, utilize a vascularized pedicled nasoseptal flap (Hadad-Bassagasteguy flap).

In summary, our findings suggest that in the hands of experienced surgeons, repeat ETS represents a safe and effective treatment option for managing residual or recurrent Cushing’s disease.

## Strengths and limitations

Our study represents one of the largest case series in the literature examining the safety, feasibility, and efficacy of repeat ETS for managing recurrent or residual CD. Our findings underscore the safety and efficacy of repeat ETS in experienced centers, showcasing satisfactory remission rates and minimal complications. However, it is important to acknowledge the retrospective nature of our study, which inherently introduces potential biases such as selection bias. Lastly, our study exclusively focuses on patients undergoing surgical intervention for recurrent or residual CD, limiting our ability to compare the effectiveness of surgical treatment with alternative modalities like radiotherapy or radiosurgery.

## Conclusion

Our study underscores the efficacy and safety of repeat endoscopic transsphenoidal surgery in managing residual or recurrent Cushing’s disease. Remarkably, 82.1% of patients achieved remission after their first reoperation, aligning closely with reported remission rates following primary endoscopic transsphenoidal surgery. Furthermore, the complication rates observed in our cohort were consistent with documented rates for both primary and repeat transsphenoidal surgeries. Notably, patients with serum cortisol levels < 5 µg/dL are more likely to maintain remission. Overall, our findings emphasize that in the hands of experienced surgeons, repeat endoscopic transsphenoidal surgery emerges as a reliable and safe treatment modality for residual or recurrent Cushing’s disease, offering satisfactory remission rates and minimal complications.

### Electronic supplementary material

Below is the link to the electronic supplementary material.

Supplementary Material 1

## Data Availability

No datasets were generated or analysed during the current study.

## Abbreviations

ACTH: adrenocorticotropic hormone
CD: Cushing’s disease
CT: computed tomography
DI: diabetes insipidus
ETS: endoscopic endonasal transsphenoidal surgery
MRI: magnetic resonance imaging
MTS: microscopic transsphenoidal surgery
