# Supplementary material for: Repeat endoscopic endonasal transsphenoidal surgery for residual or recurrent Cushing’s disease: safety, feasibility, and success
Source: Pituitary. 2024 May 15;27(3):259–68. doi: 10.1007/s11102-024-01396-x (PMC11150181; doi:10.1007/s11102-024-01396-x)
Supplement: Supplementary file 1 — Supplementary Material 1 [file 11102_2024_1396_MOESM1_ESM.pdf]

Supplementary Tables

Table 1. Patient demographics

Variable	Value
Patients	56
Sex	
Female	48 (85.7)
Male	8 (14.3)
Age, years	37.61 ± 12.41
Follow-up duration, months	97.25 ±36.87
Technique of the initial surgery	
MTS	40 (71.4)
ETS	16 (28.6)
Number of previous surgeries	
1	42 (75)
2	12 (21.4)
3	2 (3.6)
Indication for reoperation	
Residual disease	47 (72.3)
Recurrence	18 (27.7)

Values are shown as number (%) or mean ± SD unless otherwise indicated. Abbreviations: MTS, microscopic transsphenoidal surgery; ETS, endoscopic transsphenoidal surgery; ACTH, adrenocorticotropic hormone.

Table 2. Logistic regression analysis of risk factors for failed repeat ETS

Variable	Recurrence after repeat ETS	Residual disease after repeat ETS	p-value
Technique of previous surgery			
MTS	33 (64.7)	7 (50)	Reference
ETS	18 (35.3)	7 (50)	0.709
Indication for reoperation			
Residual disease	36 (70.6)	11 (78.6)	Reference
Recurrence	15 (29.4)	3 (21.4)	0.890
Tumor size			
Microadenoma	31 (60.8)	10 (71.4)	Reference
Macroadenoma	20 (39.2)	4 (28.6)	0.563
Relation to cavernous sinus			
No relationship	26 (51)	8 (57.1)	Reference
Extension	16 (31.4)	5 (35.7)	0.999
Invasion	9 (17.6)	1 (7.1)	0.999
Hardy-Wilson Classification			
Grade			
I-II	42 (82.4)	12 (85.7)	Reference
III-IV	9 (17.6)	2 (14.3)	0.577
Stage			
A-C	32 (62.7)	6 (42.9)	Reference
D-E	19 (37.3)	8 (57.1)	0.999

Values are shown as number (%). Abbreviations: MTS, microscopic transsphenoidal surgery; ETS, endoscopic transsphenoidal surgery.
